# How far are we? Assessing progress in hepatitis C response towards the WHO 2030 elimination goals by the civil society monitoring in 25 European countries, period 2020 to 2023

**DOI:** 10.1186/s12954-024-01115-6

**Published:** 2024-11-20

**Authors:** Mojca Maticic, J. Cernosa, C. Loboda, J. Tamse, R. Rigoni, E. Duffell, I. Indave, R. Zimmermann, L. Darragh, J. Moura, A. Leicht, T. Windelinckx, M. Jauffret-Roustide, K. Schiffer, T. Tammi

**Affiliations:** 1 Faculty of Medicine, University of Ljubljana, Ljubljana, Slovenia; 2 Clinic for Infectious Diseases and Febrile Illnesses, University Medical Centre Ljubljana, Ljubljana, Slovenia; 3 Correlation-European Harm Reduction Network, Amsterdam, The Netherlands; 4 European Centre for Disease Prevention and Control (ECDC), Stockholm, Sweden; 5 European Union Drugs Agency (EUDA), Lisbon, Portugal; 6 Department of Infectious Disease Epidemiology, Robert Koch Institute, Berlin, Germany; 7 Fixpunkt E. V., Berlin, Germany; 8 Free Clinic, Antwerp, Belgium; 9 Cermes3 (Inserm U988/CNRS 8211/EHESS/Université de Paris), Paris, France; 10 Finnish Institute for Health and Welfare (THL), Helsinki, Finland

**Keywords:** Hepatitis C, People who inject drugs, Continuum-of-care, Civil society, Harm reduction, Monitoring

## Abstract

**Background:**

With the advent of direct acting antivirals (DAAs) the World Health Organisation (WHO) adopted global strategy to eliminate hepatitis C virus (HCV) infection by 2030. In Europe, people who inject drugs (PWID) account for the majority of new cases, however testing and treatment remain suboptimal. The aim was to monitor progress in HCV policy and cascade-of-care for PWID, led by the civil society organisations (CSO) that provide harm reduction services for PWID across Europe.

**Methods:**

In period 2020–2023, CSOs representing focal points of Correlation-European Harm Reduction Network were annually invited to complete online questionnaire on use/impact of HCV test-and-treat guidelines for PWID, availability/functioning of continuum-of-care, and role/limitations of harm reduction services for PWID. A retrospective longitudinal analysis of responses to questions answered each year by the same respondents was performed, and a comparison among the studied years was made.

**Results:**

Twenty-five CSOs from cities in 25 European countries were included and responded to 25 questions. Between 2020 and 2023, there was positive trend in number of HCV treatment guidelines, separate guidelines for PWID, and their positive impact on acess to testing/treatment (24/25, 5/25, and 16/25 in 2023, respectively). DAAs were available in all countries, predominantly prescribed by specialist physicians only (slight increase at primary care), with restrictions including active drug use, stage of liver fibrosis or/and reimbursement policies (2/25, 4/25, and 3/25 in 2023, respectively). A decrease in HCV testing sites was noted. Treatment was consistently most common at clinical settings, however an increase outside the specialist settings was detected, particularly in prisons (12/25 and 15/25 in 2020–2021, respectively). Comparing 2022–2023, number of HCV-testing services increased in many cities with positive dynamic in nearly all the settings; increase in treatment at harm reduction services/community centres was noted (6/25 to 8/25, respectively). Between 2020 and 2023 the frequency of various limitations to CSOs addressing HCV was oscillating, presenting an increase between 2022 and 2023 (9/25 to 14/25, respectively).

**Conclusion:**

The overall progress towards WHO HCV elimination goals across Europe remains insufficient, most probably also due to the influence of Covid-19 pandemic. Further improvements are needed, also by including CSOs for PWID in continuum-of-care services, and in monitoring progress.

## Background

Hepatitis C became a key issue in harm reduction policies in the early 1990s, driven by a better understanding of its transmission, especially among people who inject drugs (PWID) [1]. Identified in 1989, the chronic nature of hepatitis C virus (HCV) infectionand severe health impacts, such as liver cirrhosis and liver cancer, led to increased focus on harm reduction policies [2, 3]. This has included promoting safer injection practices and providing clean needles and syringes through exchange programs, later adding HCV screening and treatment services for high-risk populations [4]. Beside simple and non-invasive diagnostics, the introduction of direct-acting antivirals (DAAs) in the mid-2010s turnedhepatitis C into a curable disease with their high efficacy, safety,shorter durations, and oral use [5]. These medications increased treatment uptake among key populations, including PWID, and with a cure rate of more than 95% the possibility to eliminate HCV became a reality. Indeed, in 2016, with the advent of DAAs the World Health Organization (WHO) set the first Global Health Sector Strategy (GHSS) with ambitious targets for HCV elimination by 2030, aiming to reduce new HCV infections by 90% and HCV-related deaths by 65% [6, 7].

However, hepatitis C remains a major global public health challenge [3]. The 2024 WHO Report indicated that despite excellent medical opportunities, an estimated 50 million people were still affected with HCV, significantly contributing to global morbidity and mortality [3]. In 2022, 244 000 deaths were reported globally due to long-term complications of chronic HCV infection including cirrhosis, end-stage liver disease and hepatocellular carcinoma [3]. Notably, worldwide, between the years 2020 and 2022, the HCV-seroprevalence among PWID has even increased from 8 to 9%, respectively, being far from the WHO GHSS target of reducing HCV prevalence among PWID to 2% by 2030 [8].

In Europe, a considerable burden of hepatitis C as well remains a challenge despite the national policies on HCV elimination are in place in many countries [9]. However, by 2022, in the WHO European Region only 29% of HCV infected individuals have been diagnosed and 9% of all infected have been treated, whereas 126,000 new HCV infections were reportedin that year [3]. According to a multiparametric evidence synthesis performed in European Union (EU)/European Economic Area (EEA) countries, an estimated 1.8 million people were infected with HCV, with the prevalence of chronic HCV infection ranging from ≤ 0.1 to 2.3%, and an average prevalence of around 0.5% [10]. It has also been estimated that at least 35.8% of the overall prevalence was attributed to injecting drugs, making PWID remain a crucial target for hepatitis C elimination efforts [10].

In 2019, an estimated 581 000 PWID were living in the EU and Norway [11, 12] with the seroprevalence of HCV infection varying between 16% in Czech Republic to 86% in Lithuania, reflecting differences within the population of PWID among different countries [13]. Given the high proportion of hepatitis C cases associated with injecting drug use, implementing harm reduction measures is crucial to reducing HCV transmission and prevalence. Harm reduction represents a comprehensive package of evidence-based interventions with the aim to prevent major public and individual harm without necessarily stopping using drugs, thus including needle and syringe programmes, opioid agonist treatment (OAT) and naloxone to manage overdose [4]. The provision of sterile drug paraphernalia not only reduces HCV transmission but also prevents other infections among PWID [14, 15]. However, harm reduction services remain insufficient in many countries, and government service providers often require drug abstinence from PWID to access HCV treatment [16]. Besides, several studies have shown that the efficacy of DAA treatment in PWID is as effective as in the populationthat does not use drugs; moreover, HCV prevalence among PWID was proved to decrease by unrestricted and immediately accessible HCV treatment [17, 18]. However, in several European countries PWID are facing barriers in the continuum of HCV care, including inadequate availability and accessibility to HCV testing, insufficient linkage-to-care, and limited access to HCV treatment, accompanied bystigma and discrimination [9, 19].

Therefore, a strategic approach is needed to ensure testing and treatment are available and accessible for PWID without limitations, and overcome inequity which represents a corebarrier in the progress of HCV elimination [19]. To fill in this gap, civil society organisations (CSOs) play a vital role by reaching populations and areas that government and healthcare services cannot access. Therefore, CSOsare instrumental in developing and implementing effective harm reduction interventions as well as HCV care by directly engaging with PWID, understanding their needs and often serving as first-line service providers, introducing PWID into HCV continuum-of-care [20].

However, in several European countries, there is a lack of constructive collaboration between policymakers and CSOs, leading to ineffective drug policies. In the past, CSOs across EU/ EEA reported on restricted access to DAA treatment for PWID linked to ongoing drug use, lack of health insurance, stage of liver disease, as well as other barriers in accessing HCV continuum-of-care [21–23]. Those barriers may furtherlimit the potential use of DAAs as a powerful tool to prevent further spread of HCV infection.

The progress made towards the 2030 WHO HCV elimination goals needs to be carefully monitored by regular documentation of the key policies for the general population and particularly for vulnerable populations such as PWID, and of a continuum-of-care to help countries assess the gaps and find the possible solutions. Aside WHO Reports, the European Centre for Disease Prevention and Control (ECDC) has developed a monitoring tool to help EU/EEA countries, and the European Monitoring Centre for Drugs and Drug Addiction (EMCDDA) has developed an»elimination barometer« with PWID-specific monitoring and evaluation framework, yet the major problem of all monitoring reports is the lack of appropriate data [3, 11–13, 19]. To fill in this gap, a CSO-led monitoring of HCV policies and the HCV continuum-of-care was introduced in 2019 as a complementary tool by the Correlation-European Harm Reduction Network (C-EHRN) [23].

C-EHRN is a European civil society network and centre of expertise in the field of drug use, harm reduction and social inclusion with more than 180 organisational and 140 individual members in most EU Member States and surrounding countries [24]. It is hosted by the RegenboogGroep in Amsterdam, the Netherlands, and co-funded by the EU. To support the monitoring of progress towards the WHO elimination targets at the European level, in 2019 a prospective cross-sectional survey collecting experiences of CSOs providing harm reduction services was performed by C-EHRN as a pilot for a novel complementary monitoring tool to be performed annually across Europe [23]. This tool assesses the availability and access to interventions forming the HCV continuum-of-care for PWID. The analysis of initial surveillance results in 2019 pointed to significant gaps and urged further action, as despite progress reported from several countries, further improvements were needed to the existing cascade-of-care interventions for PWID, especially as in 2019 17.1% of countries reported having no guidelines on HCV treatment and in 26.3% active drug use presented a restriction for HCV treatment [23].

The aim of this study was to analyse and compare the annual C-EHRN HCV monitoring results for the period 2020 to 2023 and examine the progress made over the past four years in the availability of interventions as part of the HCV continuum-of-care for PWID across European countries.

## Materials and methods

Based on the 2019 pilot survey on HCV monitoring in European countries [25], this is a longitudinal study that analyses retrospectively the selected results of four C-EHRN surveys in a row, in the period from 2020 to 2023, and compares them accordingly. All the surveys were prepared, conducted and analysed in the same manner through the evaluation process and rounds of consultations with the input of the C-EHRN Hepatitis C Study Group. This is an international interdisciplinary team of advisers including public health specialists, clinicians, epidemiologists, sociologists and CSO managers.

Over the study period 2020–2023, the questionnaire has slightly changed, however the majority of questions were left intact during the whole period and allowed comparison of answers among the four studied years. In the first year the questions were focused on the situation on the national level including the city level, while in the following years the focus was kept on the city/region level which allowed more precise and accurate information.

### Data collection

The respondents invited to be included in the monitoring were CSOs serving as C-EHRN focal points (FPs) in different European countries, the number of invitations varying among the studied years. Over the studied period 2020–2023, up to 40 FPs in the cities from up to 36 different countries were invited to respond to the survey. For the purpose of this study only the FPs that responded in all four years were included. Of note, due to autonomous system for HCV management, the responses of FP coming from Scotland were treated separately from the responses of FPs coming from the rest of the UK.

By definition, the C-EHRN FPs are C-EHRN member organisations and serve as the national reference points for the collection of data and information. Beside their willingness to commit to the principles of the C-EHRN, to become FPs they need to fulfil certain criteria such as proven expertise in the field of drug use and harm reduction, relevant experience in national and international cooperation, and the ability to fulfil the role of intermediary on national level [24]. For the purpose of HCV monitoring during the period 2020–2023, the selection of invited FPs was based on the C-EHRN member assessment for the pilot in 2019 [23], followed by including additional FPs or omitting some of them, depending on their activity and participation.

Each of the studied years in the period 2020–2023, the participating FPs were invited to join the survey by completing the online questionnaire—one questionnaire was completed per city. Each year the responses were collected by C-EHRN and reviewed by the C-EHRN Hepatitis C Study Group. In case of incomplete, unclear or inconsistent responses, the respondents were given additional questions via email for re-checking. If the information given by repeated email responses remained unclear, respondents were contacted by phone to obtain a clarification and/or to validate the meaning of the response.

### The questionnaire

Each of the four online questionnaires, designed for the purpose of the surveys over the observational period 2020–2023, consisted of 25 up to 27 questions based on the previous C-EHRN experiences and the external expert input, mainly gathered from the practitioners in the field [25]. Namely, in 2018 the C-EHRN established thematic study groups, each consisting of six experts that were invited to contribute on a voluntary base to the different C-EHRN activities. Each year, there were slight changes made in the questionnaire, thus the HCV Study Group was asked to review the proposed questionnaire, particularly regarding the monitoring questions, and to contribute with comments, data analysis and/or final monitoring report. Additionally, this process was supported also by the researchers outside the C-EHRN, such as ECDC and EMCDDA who helped developing, adapting and reviewing the monitoring activities of C-EHRN as part of its Scientific Advisory Board.

Following the pilot survey from 2019 [23], each of the questionnaires in the observational period 2020 to 2023 addressed four strategic fields: the use and impact of national strategies and guidelines on accessibility to HCV testing and treatment for PWID; the availability and functioning of the continuum-of-care in different countries/cities; potential changes in thecontinuum of services compared to the previous year; and, the role of harm reduction servicesand civil society organisations in this context.

The term PWID, used in the surveys during the observational period includes three different groups of individuals:»active PWID« referring to those who had injected drugs within the past six months [26];»PWID on opioid agonist treatment (OAT) « referring to those who were currently included in an OAT programme (either quitted injecting or still occasionally injecting drugs); and»former PWID« referring to those who completely stopped injecting drugs and were not using OAT.

The answers to the majority of questions were binary (»yes«/»no«); however, there were multiple choice answer options in some questions. Besides, a free-text box offered the respondents to add comments for clarifying the given information or provide additional qualitative information, links or other sources. The questionnaires were administered in English only because language barriers were not expected.

### Study design

For the purpose of this study the responses from all four surveys in the observational period 2020–2023 were included. Only the FPs that responded to all four questionnaires were included in our study; furthermore, only the questions that remained the same during the whole study period were included. The aim of this study was to analyse the questions that have beenanswered each year by the same respondents, and make a comparison of responses over the study period 2020–2023 to observe possible development achievements.

### Data analysis

A descriptive and geospatial analysis was performed. For every question, every year and all the respondents the counts summaries and frequencies were performed and analysed accordingly.

## Results

**Fig. 1:**
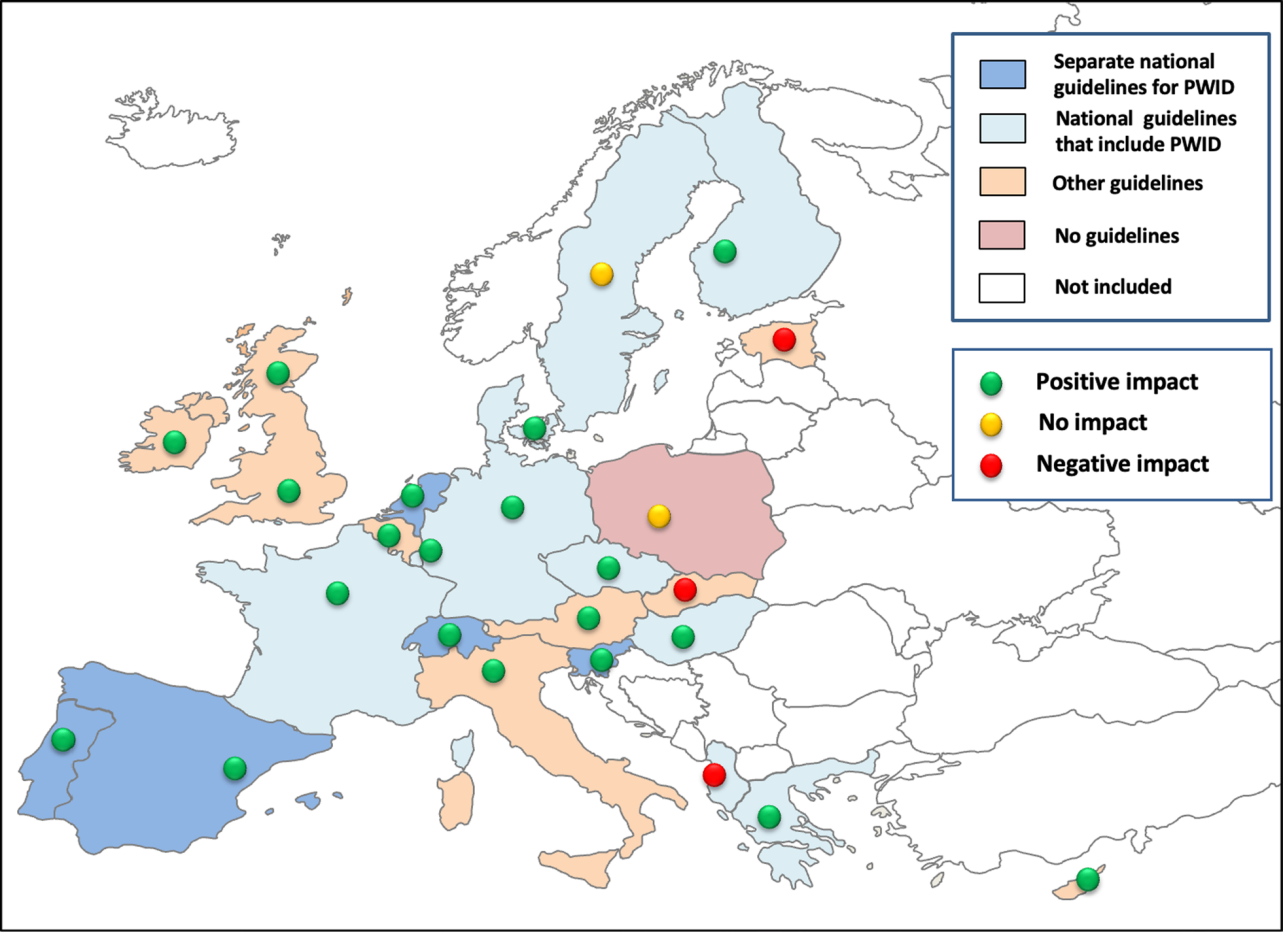
Twenty-five European countries included in the study, and the reported use of the most relevant guidelines for hepatitis C management including their perceived impact in 2023. ^#^Scotland was treated separately from the rest of the UK. ^##^The countries in white did not participate in the study. PWID—people who inject drugs

Twenty-five FPs coming from the cities in 25 different European countries were included (Fig. 1) and responses to 25 questions were analysed, covering all four strategic fields.

Since 2022 on, the respondents were asked whether they consulted other experts before answering the questionnaire. In 2022, 18/25 respondents (72%) did so, while in 2023 this number increased to 19/25 (76%) with no answer received from one city (4%).

### The progress in guidelines for treatment of hepatitis C in people who inject drugs

**Fig. 2:**
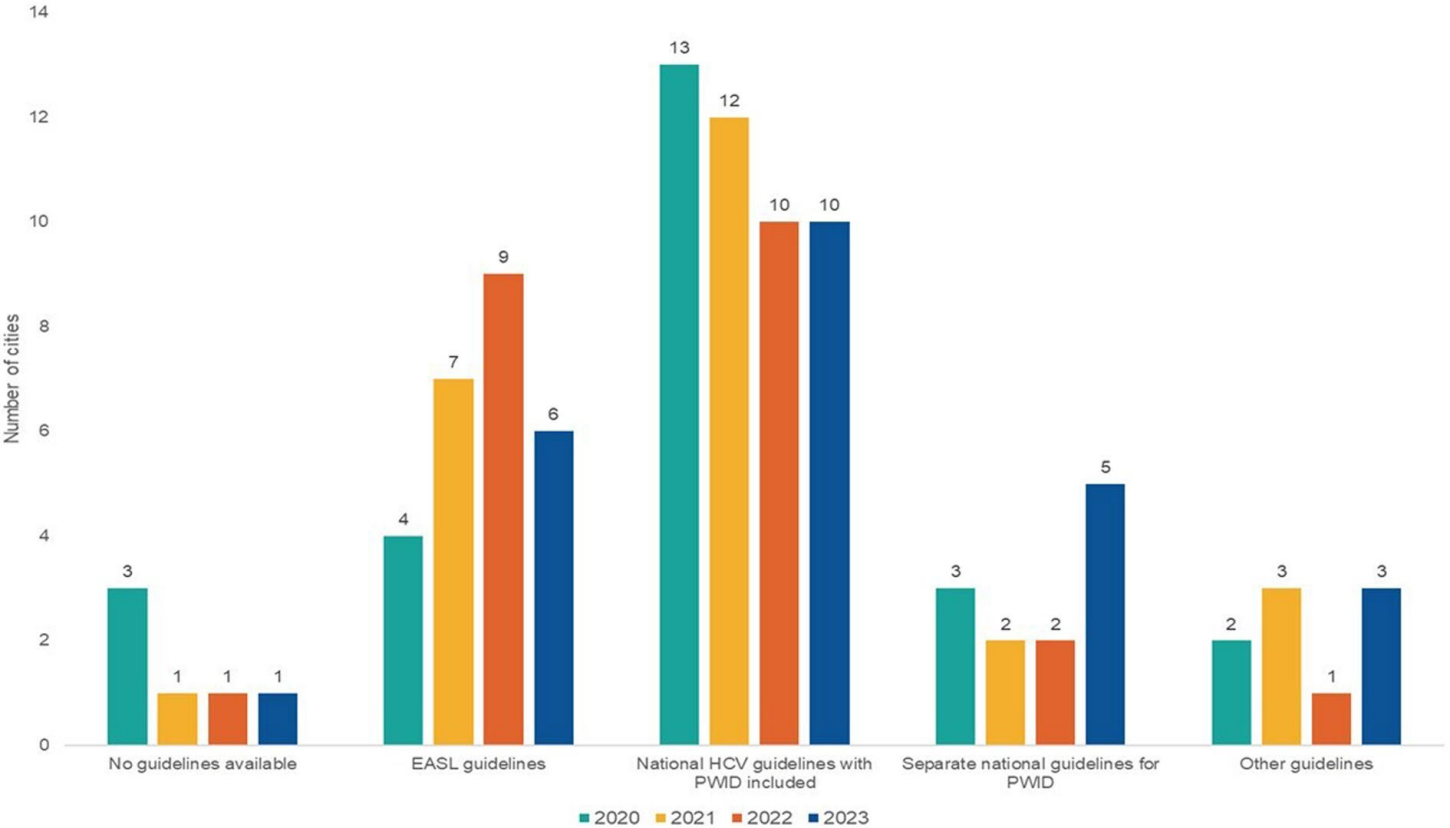
The most relevant guidelines for hepatitis C treatment in people who inject drugs in 25 European countries, period 2020 to 2023. EASL—European Association for the Study of the Liver, HCV—hepatitis C virus, PWID—people who inject drugs

The most relevant guidelines that were used for HCV treatment of PWID reported from 25 included countries and their perceived impact on accessibility to testing and treatment in the year 2023 are presented in Fig. 1. The changes in the use of guidelines for hepatitis C treatment in PWID over the observational period are shown in Fig. 2.

Across the 25 cities, the presence of separate national guidelines for HCV treatment of PWID remained limited with most cities not reporting having them throughout the observational period. In 2020 only 3/25 (12%) FPs reported on having separate guidelines, with the number increasing to 5/25 (20%) in 2023: The Netherlands, Portugal, Slovenia, Spain, and Switzerland. Denmark initially had separate guidelines for PWID in 2020 but transitioned to national guidelines that included PWID in subsequent years. Slovakia reported on using separate guidelines from 2020 to 2022 and switching to other guidelines in 2023. As for the rest of the countries, in 2020 13/25 (52%) FPs reported having the national guidelines that included PWID, with the number decreasing to 12/25 (48%) in 2021, and further to 10/25 (40%) in 2022 and 2023; no guidelines for treating HCV-infected PWID were in 2020 reported from 3/25 FPs (12%), while by 2023 only one country (4%), Poland, reported on having no such guidelines.

Of the respondents that reported on having some kind of guidelines (24/25, 96%), the majority presented a positive impact of the guidelines on testing and treatment accessibility across all years. In 2023, all countries presenting separate national guidelines for PWID reported their positive impact (5/5, 100%), while 8/10 (80%) FPs presenting the national guidelines that included PWID, and 7/9 (78%) FPs presenting other guidelines reported their positive impact on testing and treatment accessibility; 1/10 (10%) FPs reported on a negative impact of the national guidelines (Tirana), and 1/10 (10%) reported on no impact (Stockholm), whereas among nine FPs presenting other guidelines, 2/9 (22.2%) FPs reported a negative impact (Bratislava, Tallinn), and one FP reported no guidelines (Warsaw) (Fig. 1).

The positive impact of guidelines reported from 20/24 (83%) cities means, that from the year 2020 to the year 2023, the number of cities reporting access of PWID to CSO increased from 8/20 (40%) to 13/20 (65%), the reported access to specialized HCV services increased from 12/20 (60%) to 17/20 (85%), and the reported access to information and counselling due to guidelines increased from 13/20 (65%) to 19/20 (95%) cities. An improvement was observed also in the impact of guidelines on accessibility to HCV testing and treatment, increasing from 11/20 (55%) and 13/20 (65%) in 2020, respectively, to 19/20 (95%) and 20/20 (100%) in 2023, respectively. However, 3/24 (13%) FPs reported some negative impact, with Bratislava being the only city reporting a negative impact of the guidelines across all observational years. Additionally, there were some reports of no impact from the guidelines, with the highest number occurring in 2021 (3/24, 13%).

**Fig. 3:**
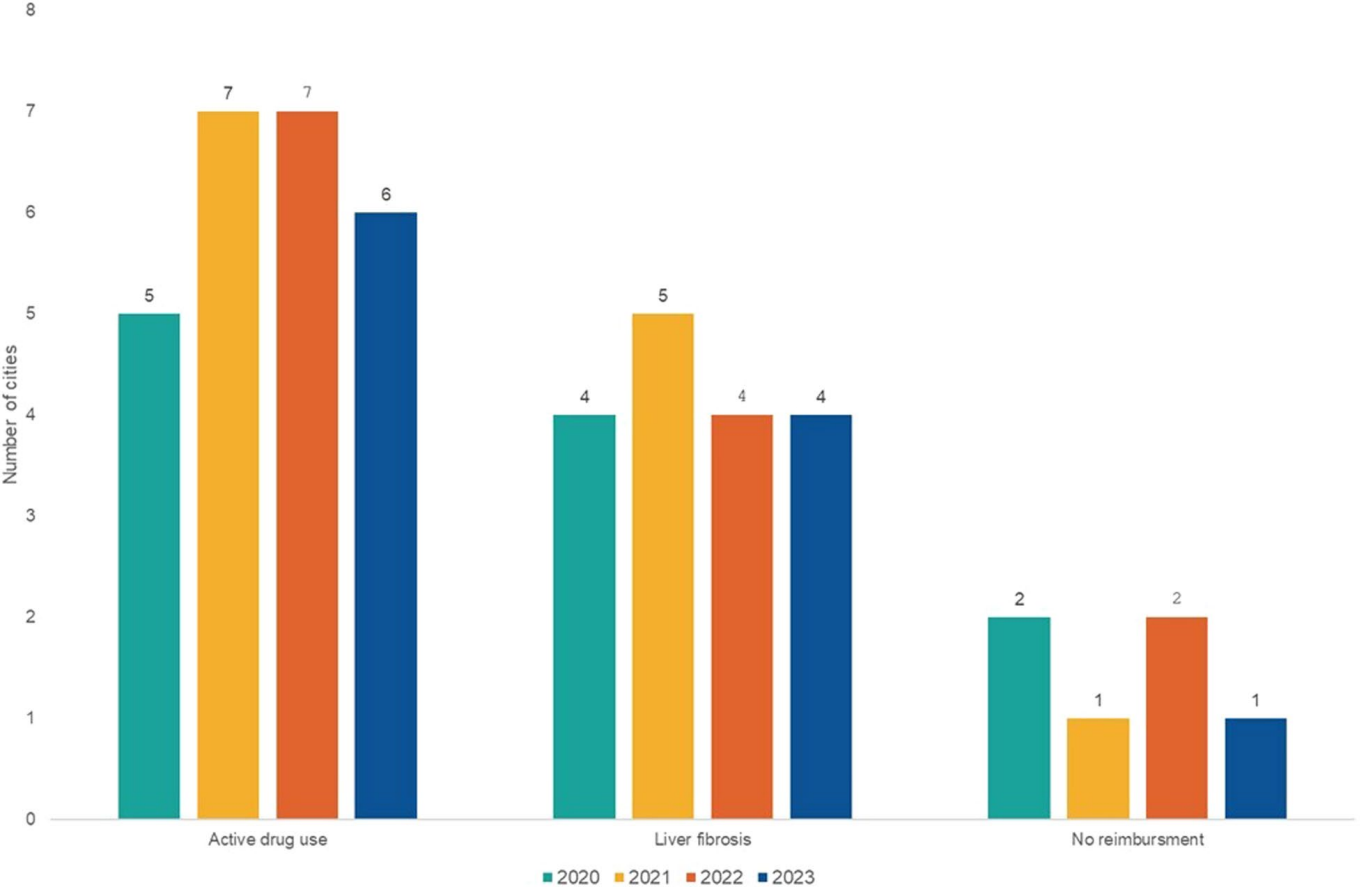
Reported restrictions to treatment with direct acting antivirals in 25 European cities, period 2020 to 2023. DAA—direct acting antivirals

According to the respondents, DAAs were available in all reporting countries (25/25, 100%) across all the reporting years. However, over the four observational years, some countries reported restrictions to their use for PWID, or due to stage of liver disease or lack of reimbursemet for DAAs (Fig. 3). Unrestricted use of DAAs was consistently reported only by 15/25 (60%) respondents.

Restrictions on the use of DAAs for PWID were reported by 5/25 (20%) FPs in 2020, 7/25 (28%) in 2021 and 2022, and 6/25 (24%) in 2023, respectively (Fig. 3, Table 1). Respondents from Bratislava, Copenhagen and Tirana consistently reported restrictions across the whole observational period. Specifically, in Bratislava a 12-months abstinence from drugs is required and needs to be confirmed every three months by toxicological examinations as a condition for DAA treatment. On the other hand, Milan and Vienna reported newly introduced restrictions on DAAs in the last observational year, while Nicosia and Amsterdam reported a lifting of restrictions in 2023. There were no reported restrictions for PWID on OAT, or people formerly injecting and not being on OAT from any of the included countries.

**Table 1:** The availability of hepatitis C continuum-of-care in harm reduction services and community centres for people who inject drugs

	Free HCV testing				POC HCV screening				Confirmatory HCV RNA testing				Written protocol/ guidelines for linkage-to-care				Non-invasive liver fibrosis assessment				DAA treatment				Legal prescription of DAAs by GPs/ nurses/ pharmacists				DAAs for PWID without restrictions			
	2020	2021	2022	2023	2020	2021	2022	2023	2020	2021	2022	2023	2020	2021	2022	2023	2020	2021	2022	2023	2020	2021	2022	2023	2020	2021	2022	2023	2020	2021	2022	2023
Albania Tirana	na	na	N	N	Y	Y	Y	na	N	N	N	N	na	na	na	N	N	N	N	N	N	N	N	N	N	N	N	N	N	N	N	N
Austria Vienna	na	na	Y	Y	N	N	N	na	N	N	N	Y	N	N	N	N	N	N	N	N	N	N	N	N	N	N	N	N	Y	Y	Y	N
Belgium Antwerpen	na	na	Y	Y	Y	Y	Y	na	N	Y	Y	Y	Y	N	N	na	N	N	Y	Y	N	Y	N	N	N	N	N	N	Y	N	Y	Y
Cyprus Nicosia	na	na	N	N	Y	Y	Y	na	N	N	N	N	N	N	N	N	N	N	N	N	N	N	N	N	Y	Y	N	N	N	N	N	Y
Czechia Prague	na	na	Y	Y	Y	Y	Y	na	Y	Y	Y	Y	Y	Y	Y	Y	Y	Y	N	N	Y	Y	N	Y	Y	N	N	N	Y	Y	Y	Y
Denmark Copenhagen	na	na	na	N	Y	Y	Y	na	Y	Y	Y	Y	na	na	na	N	Y	Y	Y	Y	N	N	N	Y	N	N	N	N	N	N	N	N
England London	na	na	Y	Y	Y	Y	Y	na	Y	Y	Y	Y	Y	Y	Y	N	Y	Y	Y	Y	Y	Y	Y	Y	Y	Y	Y	Y	Y	Y	Y	Y
Estonia Tallinn	na	na	Y	N	Y	Y	Y	na	N	N	N	N	na	Y	Y	Y	N	N	N	N	N	Y	N	N	N	N	N	N	N	N	Y	N
Finland Helsinki	na	na	Y	Y	Y	Y	Y	na	Y	Y	Y	Y	N	Y	Y	Y	N	N	N	N	Y	Y	Y	Y	N	Y	Y	Y	Y	Y	N	Y
France Paris	na	na	Y	Y	Y	Y	Y	na	Y	Y	N	Y	na	Y	na	Y	Y	Y	Y	Y	Y	Y	Y	Y	Y	Y	Y	Y	Y	Y	Y	Y
Germany Berlin	na	na	na	Y	Y	Y	Y	na	Y	Y	Y	Y	Y	Y	Y	N	N	N	N	N	N	N	Y	N	Y	Y	Y	Y	Y	Y	Y	Y
Greece Athens and Tessaloniki	na	na	Y	Y	Y	Y	Y	na	N	N	N	N	N	N	N	N	Y	N	N	N	N	N	N	N	N	N	N	Y	Y	Y	Y	Y
Hungary Budapest	na	na	N	N	Y	Y	Y	na	N	N	N	N	N	N	N	N	N	N	N	N	N	N	N	N	N	N	N	N	Y	Y	Y	Y
Ireland Dublin	na	na	Y	Y	Y	Y	Y	na	Y	Y	Y	Y	N	na	na	N	Y	N	N	N	N	N	N	N	N	Y	Y	Y	Y	Y	Y	Y
Italy Milano	na	na	N	N	Y	Y	Y	na	N	N	N	N	N	N	N	Y	N	N	N	N	N	N	N	N	N	N	N	N	Y	Y	N	N
Luxembourg	na	na	Y	Y	Y	Y	Y	na	Y	Y	Y	Y	Y	N	N	Y	Y	Y	Y	Y	Y	Y	Y	N	N	N	Y	N	Y	Y	Y	Y
Poland Krakow	na	na	Y	Y	Y	Y	Y	na	N	N	N	N	N	N	N	Y	N	N	N	N	N	N	N	N	N	N	N	N	Y	Y	Y	Y
Portugal Porto	na	na	Y	Y	Y	Y	Y	na	Y	Y	Y	Y	N	N	N	Y	N	N	N	N	Y	Y	Y	Y	N	N	N	N	Y	Y	Y	Y
Scotland Glasgow	na	na	Y	Y	Y	Y	Y	na	Y	Y	Y	Y	Y	Y	Y	Y	N	N	N	N	N	Y	N	Y	N	Y	Y	Y	Y	Y	Y	Y
Slovakia Bratislava	na	na	N	N	Y	Y	Y	na	N	N	N	N	na	na	na	N	N	N	N	N	N	N	N	N	N	N	N	N	N	N	N	N
Slovenia Ljubljana	na	na	Y	Y	Y	Y	Y	na	Y	Y	Y	Y	na	na	na	Y	N	N	N	N	N	N	N	N	N	N	N	N	Y	Y	Y	Y
Spain Barcelona	na	na	N	Y	Y	Y	Y	na	Y	Y	Y	Y	Y	Y	Y	Y	Y	Y	Y	Y	Y	Y	Y	Y	N	N	Y	N	Y	Y	Y	Y
Sweden Stockholm	na	na	Y	Y	Y	Y	Y	na	Y	N	N	N	Y	N	N	N	Y	N	N	N	Y	N	N	N	N	N	N	N	Y	Y	Y	Y
Switzerland Bern	na	na	N	N	Y	Y	Y	na	N	N	Y	Y	N	N	N	N	Y	Y	N	N	Y	Y	N	N	N	N	Y	Y	Y	Y	Y	Y
The Netherlands Amsterdam	na	na	Y	Y	Y	Y	Y	na	N	Y	N	N	Y	Y	Y	Y	N	N	N	N	N	N	N	N	N	N	Y	N	Y	N	N	Y

*Y* Yes, *N* No, *na* Data not available, *HCV* Hepatitis C virus, *RNA* Ribonucleic acid, *POC* Point-of-care, *DAA* Direct-acting antivirals, *GP* General practitioner, *PWID* People who inject drugs

The reported restrictions for DAA treatment included also the stage of liver fibrosis which has been reported from 4/25 (16%) countries with the exception of the year 2021 (5/25, 20%) (Fig. 3). Regarding the reimbursement for DAA treatment, in 2020, 20/25 (80%) cities reported unrestricted reimbursement for DAAs, while 2/25 (8%) cities reported no reimbursement at all. By 2021, the number of cities offering full reimbursement increased to 22/25 (88%) and remained unchained until 2023. Throughout the whole observational period, FPs from Bratislava and Prague consistently reported on reimbursement for DAAs with limitations, while FP from Tirana reported on no reimbursement for DAA treatment at all. In contrast, FPs from London and Stockholm reported on improving reimbursement status in 2021 and further on offer reimbursement without limitations.

### The progress in the continuum-of-care for hepatitis C management in people who inject drugs

A continuum-of-care that includes screening and confirmatory testing, linkage-to-care, assessment of the severity of liver disease and HCV treatment with DAAs has been performed in a wide range of services for PWID across Europe. The availability of continuum of services for HCV management in harm reduction services and community centres is presented in Table 1.

Screening tests to detect anti-HCV antibodies either in saliva (oral fluid swabs) or blood (finger prick) were available free-of-charge for PWID in 16/25 (64%) cities in 2022, and increased to 17/25 (68%) cities in 2023. Over the whole observational period, free of charge testing was consistently offered in 15/25 (60%) cities (Table 1). Point-of-care (PoC) antibody testing was predominantly available at harm reduction services/community centres in the first three observational years (24/25, 96%), and Vienna was reported to be the only city without availability of free PoC anti-HCV testing; no data was available for the year 2023 (Table 1). In prison settings it was available in 15/25 (56%) cities in 2022 with little variation over the reporting period. At infectious diseases clinics PoC testing was performed in 16/25 (64%) cities in 2020 with some decrease in 2022 (14/25, 56%). Similarly, the availability of PoC testing reported at drug treatment clinics was present in 16/25 (64%) cities in 2020 and 2022, with a slight drop to 15/25 (60%) in 2021, while at gastroenterology clinics it was available in 11/25 (44%) in 2020, and decreased to 9/25 (36%) by 2022. The lowest availability of PoC anti-HCV testing was reported at general practitioners (12/25, 48% in 2020; 11/25, 44% in 2022) and in pharmacies (4/25, 16%) in 2020; 3/25, 12% in 2022).

**Fig. 4:**
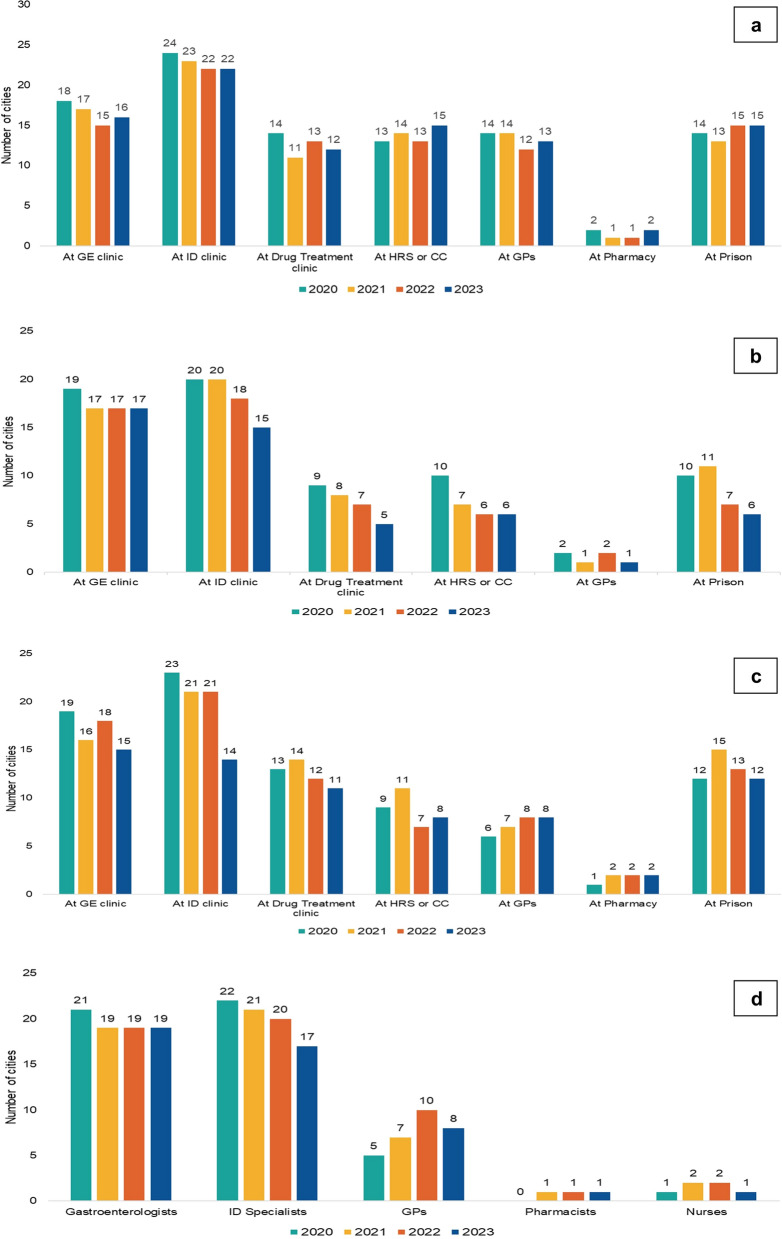
The continuum-of-care for managing hepatitis C in people who inject drugs in 25 European cities, period 2020–2023. **a** The number of cities offering confirmatory testing at different settings. **b** The number of cities offering non-invasive diagnostic procedures for liver disease assessment at different settings. **c** The number of cities offering treatment at different settings. **d** The number of cities with a legal prescription of direct acting antivirals by different healthcare providers. ID—infectious diseases, GE—gastroenterology, GPs—general practitioners, HRS—harm reduction services, CC—community centres

Confirmatory HCV RNA testing (Fig. 4a) was most reported at infectious diseases clinics in 2020 (24/25, 96%) with some decrease noted in 2023 (22/25, 88%), while for gastroenterology clinics it was reported from 18/25 (72%) cities in 2020, but was reported from 16/25 (64%) cities in 2023. The availability of confirmatory HCV RNA testing at drug treatment clinics varied a little over time from14/25 (56%) cities in 2020 to 11/25 (44%) in 2021. In prisons its availability was reported from 14/25 (56%) cities in 2020, with little changes over the reporting period. At harm reduction services/community centres the confirmatory testing was reported to be available in a little more than 50% across all observational years, while at general practitioners it was the highest in 2020 (14/25, 56%) and the lowest in 2022 (12/25, 48%) (Table 1). The availability of confirmatory HCV RNA testing was the lowest in pharmacies (Fig. 4a), and was only available in London across the whole reporting period, and for 2020 and 2023 in Glasgow (Fig. 4a, Table 1).

In 2020, 2021 and 2022, 9/19 (47%), 9/20 (45%) and 8/19 (42%) FPs, respectively, reported on having a written protocol or guidelines facilitating linkage-to-care for PWID. In 2023, there was a notable improvement, as 12/24 (50%) cities reported the implementation of such protocols, however, the status remained unknown in one city, Antwerp (1/25, 4%) (Table 1).

In 2020, the non-invasive liver fibrosis assessment (Fig. 4b) was reported to be available at gastroenterology clinics in 19/25 (76%) cities, declining to 17/25 (68%) in 2021 and remaining constant thereafter. Non-invasive liver fibrosis assessment was provided at infectious diseases clinics in 20/25 (80%) cities in 2020/2021, decreasing to 15/25 (60%) cities in 2023. In 2020, drug treatment clinics were reported to offer non-invasive liver fibrosis assessment in only 9/25 (36%) cities, which decreased to 5/25 (20%) in 2023. Similarly, a decline was noted also at harm reduction settings/community centers (from 10/25, 40% in 2020, to 6/25, 24% in 2023) (Table 1), at GPs (2/25, 8% in 2020, to 1/25, 4% in 2023), and prisons (from 10/25, 40% in 2020, to 6/25, 24% in 2023) (Fig. 4b).

According to respondents, the DAA treatment (Fig. 4c) was mostly initiated at infectious diseases clinics (23/25, 92% in 2020, decreasing to 14/25, 56% by 2023), gastroenterology clinics (19/25, 76% in 2020, decreasing to 15/25, 60% by 2023), prisons (12/25, 48% in 2020, increasing to 15/25, 60% in 2021, and decreasing to 12/25, 48% by 2023), and harm reduction services/community centres (9/25, 36% in 2020, increasing to 11/25, 44% in 2021, and decreasing to 8/25, 32% by 2023) (Table 1). The lowest availability of DAA treatment was reported at general practitioners (6/25, 24% in 2020, increasing to 8/25, 32% by 2023), and pharmacies (1/25, 4% in 2020, increasing to 2/25, 8% by 2023) (Fig. 4c).

According to the responses analysed, the prescription of DAA therapy (Fig. 4d) was most commonly recorded by infectious diseases specialists (22/25, 88% in 2020, with a decrease to 17/25, 68% by 2023) and gastroenterology specialists (21/25, 84% in 2020, with a decrease to 19/25, 76% by 2023). General practitioners prescribed DAAs in 5/25 (20%) cities in 2020, increasing to 10/25 (40%) in 2022, and 8/25 (32%) in 2023 (Table 1, Fig. 4d). In 2020, nurses and pharmacists were not able to prescribe DAAs. However, since 2021, pharmacists have been able to prescribe DAAs in one (4%) city, while nurses were allowed to prescribe DAAs in 2/25 (8%) cities in 2021 and 2022, which dropped back to one city (4%) in 2023 (Fig. 4d).

### The dynamic of limitations to harm reduction organizations addressing hepatitis C

**Fig. 5:**
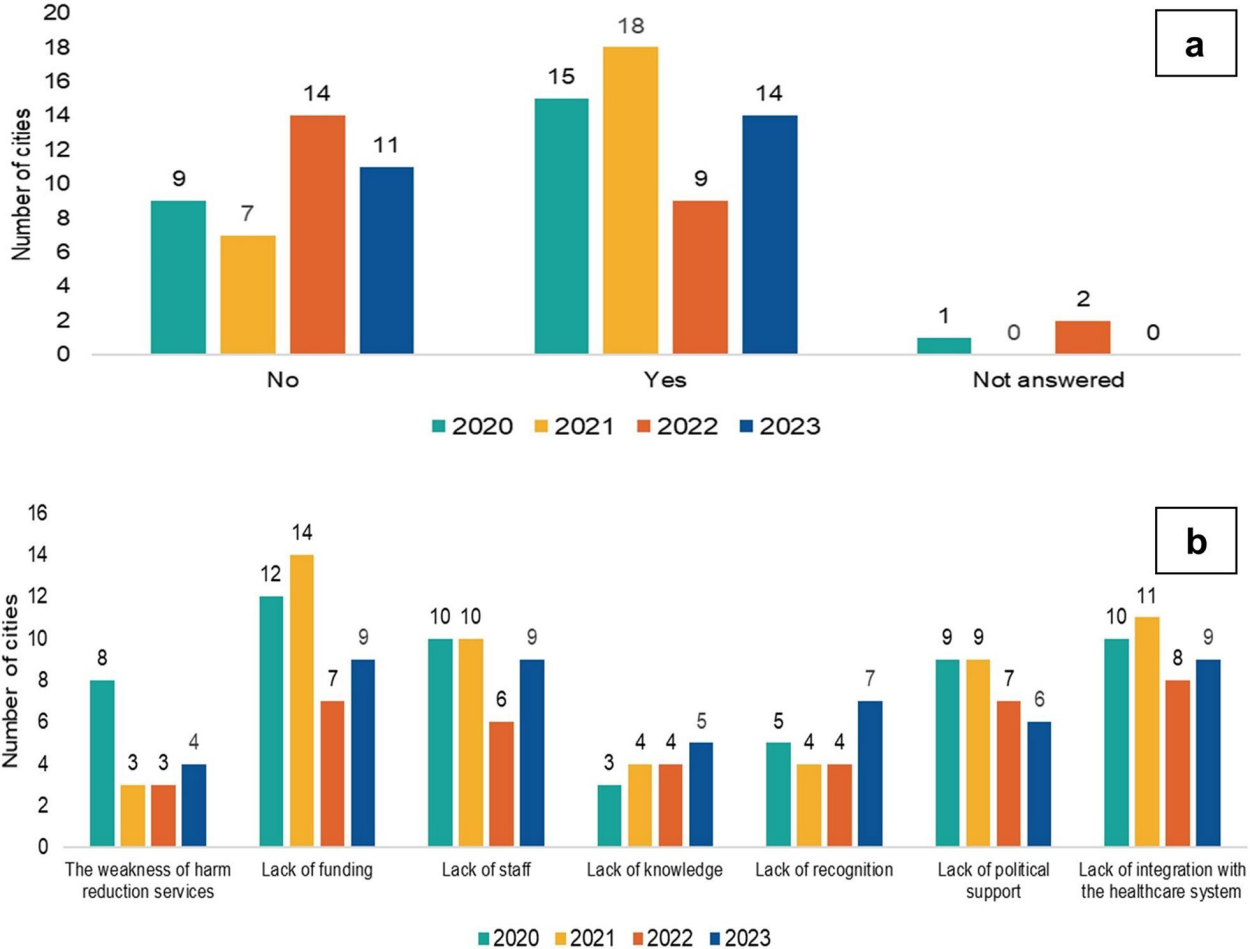
Harm reduction organisations addressing hepatitis C in 25 European cities, period 2020–2023. **a** The number of cities reporting on limitations or lack of limitations in addressing hepatitis C at harm reduction organisations. **b** Perceived specific barriers to address hepatitis C at harm reduction organisations. No—no limitations, Yes—limitations are present

The number of FPs reporting limitations to harm reduction organisations in addressing HCV varied during the observational period (Fig. 5a). In 2020, a minority of FPs (9/25, 36%) reported no limitations to harm reduction organisations in addressing HCV, but15/25 (60%) FPs reported limitations. Over the years 2021 to 2023 variations in the presence or absence of limitations were noted, with the increase in the reported limitations between the last two observational years from 9/25 (36%) to 14/25 (56%), respectively (Fig. 5a). The limitations that were identified and reported by FPs as major concerns in addressing HCVat harm reduction organisations were lack of funding, weakness of harm reduction, lack of knowledge, lack of recognition, lack of political support, lack of integration with the healthcare system and lack of staff. The proportions of perceived barriers reported from FPs are presented in Fig. 5b.

In 2023, an additional question on stigma and discrimination against PWID in HCV care facilities was included, together with a question on monitoring and management of the latter. 24/25 FPs responded, while data from Stockholm was missing. The respondents from two cities (Glasgow and Vienna) reported stigma and discrimination in all HCV care settings, while five cities (Helsinki, Krakow, Ljubljana, Luxembourg, Paris) reported no such incidents being detected. Gastroenterology clinics, primary centres and prisons were most frequently cited as places where stigma and discrimination were reported (13/24 each, 54%), followed by pharmacies (12/24, 50%), infectious diseases clinics (11/24, 46%), drug treatment clinics (7/24, 29%), and lastly harm reduction services/community centres (3/24, 13%). Only 6/24 (25%) FPs reported monitoring and management of stigma/discrimination incidents, while no action of the kind was reported from 15/24 (62.5%) cities; 3/24 (13%) FPs could not provide a consistent response.

## Discussion

To the best of our knowledge, this is the first study on the progress in response to the HCV epidemic in PWID towards HCV elimination from a real-life perspective of CSOs across Europe over a four-year period that spanned the period of the Covid-19 pandemic.

The latest WHO global data clearly revealed, that in this decade, viral hepatitis remains a major public health challenge, and we are still far from achieving the elimination by 2030 [3]. Monitoring the progress toward the WHO elimination goals in Europe showed that hepatitis C elimination has been proceeding at different speeds, with inequities across the countries and within a particular country. To evaluate the current country situation in European countries and speed-up the processes, established monitoring systems are in place to provide updated surveillance data and follow the progress in order to recommend future directions and activities, needed in particular countries.

The WHO Global Health Sector Strategy (GHSS) on Viral Hepatitis set up in 2016 [7] has been followed by the WHO Global Reports in 2016, 2018 and 2020, and the last being launched in April 2024 [3, 27–29]. The latter was the first consolidated WHO report on HCV epidemiology, service coverage, and access to medical products. It clearly revealed, that compared to the data from 2019 Report, in the WHO European region the proportion of individuals with confirmed HCV diagnosis has increased from 24 to 29%, respectively and the proportion of treated individuals has increased by one percent only (from 8 to 9%, respectively). Consequently, the 2024 Report provides the WHO regional perspectives including the analysis of the barriers and opportunities for countries to expand their access to medical products for viral hepatitis. Besides, it brings about the recommendations for actions particularly for stakeholders in low- and middle-income countries to increase and accelerate scaling-up of effective HCV interventions [3].

In Europe, the first WHO European Region Action Plan adapted to the GHSS was set up in 2016, taken into account the epidemiological, political, and social particularities of European countries [30].

Followed in 2019, the ECDC has developed a monitoring system for hepatitis C to support 30 countries in the EU/EEA in monitoring responses to HCV epidemic towards HCV elimination by collecting data from a range of existing sources. This monitoring system has been closely aligned with the indicators and HCV elimination targets of the WHO GHSS, as well as with the WHO European Region Action Plan. The progress has been monitored in the ECDC Technical Reports from 2019, 2021 and 2023, the latter being launched in April 2024 [9, 15, 31, 32], all of them highlighting significant gaps in the availability of data related to the HCV continuum-of-care such as prevention, testing and treatment, as well the policies related to them. The 2023 report presented that out of 30 monitored countries, 29 provided national data for at least one of the key stages of the HCVcontinuum-of-care,whereas only four countries reported data along the whole continuum [9]. The third round data collection conclusion was that the HCV burden in EU/EEA countries remained high and disproportionately affected different key populations including PWID. To reach the elimination targets, a multidisciplinary approach is needed based on data collection from various comprehensive and sustainable monitoring systems.

Without any doubt, PWID represent the driving force of HCV epidemic in Europe and a key population for the elimination of hepatitis C, both in terms of transmission thus requiring higher levels of combined prevention, as well as in terms of the burden, requiring better access to testing and treatment. In 2019, the EMCDDA established an elimination barometer for hepatitis C helping EU countries, Norway and Turkey to assess their progress towards eliminating HCV among PWID [33]. This barometer has been conducting together with the drug related infectious diseases (DRID) network, as well as in close collaboration with the ECDC. The continuum of EMCDDA reports including the latest one from 2023 revealed permanent lack of information in several countries, particularly the absence of systematic data collection on HCV continuum-of-care for PWID.However, the data available clearly showed that compared to 2015, in 2021 HCV transmission among PWID remained high, pointing out the urgent need for scaling up access to integrated and stigma-free prevention and careamong PWID across Europe, and improved access to current data.

The CSOs have already proved their most valuable contribution in filling the gaps of both formal and informal monitoring systems, first being presented in the global HIV response [34]. Due to their community-based overview of the real-life situation, they can exceptionally contribute not only in disclosing the major gaps and barriers in the continuum of HCV care, but particularly in strengthening commitments to human rights and fight against stigma and discrimination.

Since the C-EHRN represents the largest civil society network in the field of drug use and harm reduction in Europe, the monitoring system covering certain areas of drug policy and practice was introduced in 2019 to enrich the information based on the perspective of CSOs working with PWID, including the information on the availability and access to interventions forming the three key stages of a HCV continuum-of-care for PWID. The analysis of the initial surveillance results in 2019 from 35 European countries pointed to significant gaps and urged further action, especially as in 2019, 17% of countries reported on having no HCV treatment guidelines, and in 26% of countries active drug use represented a restriction for HCV treatment [23]. To follow possible progress achieved in the next years, an annual monitoring was introduced, and the dynamic of data over the period 2020–2023 was analysed and presented here.

There were considerable varieties in responses given over the four studied years from 25 European cities included, located in 25 different countries, showing a Central-Western and Northern European distribution. In the last two study years, the majority of respondents revealed consultations with local experts before completing the questionnaire, confirming their credibility and responsibility towards the role and importance of this kind of monitoring.

There are many challenges in achieving HCV elimination, however one of the basic is having the possibility of highly effective and safe HCV treatment [5, 35]. During the observational period there was a positive trend in the number of cities reporting their country having HCV treatment guidelines, however the typeof the guidelines including PWID has changed over time. In 2023, despite a few countries still reporting on using the EASL guidelines, the national treatment guidelines were in place in all but one responding country. However, from the year 2020 to the year 2023, the proportion of countries reporting the inclusion of PWID in those guidelines decreased, yet the proportion of countries presenting specific national guidelines for treating PWID increased in 2023. It is worth mentioning that only a couple of countries reported on no, or even a negative impact of guidelines on the treatment accessibility for PWID, whereas more than half of them reported their positive impact across all the examined years, particularly all those presenting any kind of guidelines for PWID.

Starting with the year 2020, DAAs were reported to be available in all included countries, yet predominantly prescribed only by specialist physicians being reported over the whole observational period, with a slight increase in the number of prescribers among the general practitioners. With regard to the DAA treatment policy, the restrictions included either active drug use, stage of liver fibrosis or/and reimbursement policies. Indeed, even in 2023, DAA treatment for active drug users has still been prohibited in six included European countries. A slight progress has been made regarding the reimbursement of DAA treatment without limitations, in 2023 remaining a barrier in six of the included countries. According to the latest global data, DAA treatment has been reimbursed in 52% of 89 low- and middle-income countries, however in 6% of them active drug use represented a restriction to DAA treatment, with restrictions present also for the early liver fibrosis stages reported from 3% of the countries; of note, in 61% of the countries, a specialist prescribing DAAs was required [35, 36].

The C-EHRN monitoring data on the continuum of HCV care in Europe shows that a wide range of services have been available for PWID.However, the data on progress achieved during the observational period revealed both, the best practices and areas that still need improvement, highlighting that testing and treatment services were still provided in a limited variety of settings in several cities included.

There were considerable differences across Europe as to where and how PWID can access HCV testing, particularly regarding the settings inside and outside the healthcare system. Free-of-charge HCV testing was permanently offered in half of the included cities. While at harm reduction services and community centres PoC screening tests were available in all but one included city, the confirmatory HCV RNA testing was reported from a little more than half of them.

Conversely, in prison settings, the screening and confirmatory test availability was consistently reported, with over half of the cities reporting PoCHCV antibody, as well as HCV RNA testing being offered there. Compared to the general population, HCV infection disproportionately affects individuals in prisons, due to high levels of past/current injecting drug use among incarcerated persons, highlighting the need for preventive interventions and providing aunique setting for HCV care [37, 38].Prisons offer an opportunity for a one-stop-shop approach with test-and-treat strategy at one place including PoCtesting and nurse-led care which has been shown to increase treatment uptake as well as reduces time to treatment initiation [39]. The EASL recommendations identified incarcerated persons as a population that may benefit from simplified and streamlined interventions [5]. Despite these recommendations, globally there have been significant variations in HCV care within prisons and the cities in our study reporting the lack of such a possibility miss the opportunity to identify HCV in this high-risk population [40].

The general picture over the years 2020–2023 reveals that there has been a considerable decline in the proportion of cities offering confirmatory testing in settings, such as specialist infectologist and gastroenterologist clinics, as well as drug treatment clinics, whereas a stable or even increasing trend was observed at general practitioners, harm reduction services, community centres, and prisons. This result might refer to the influence and restrictions of the Covid-19 pandemic [41], yet it may also reflect the decentralisation trends as a strategy to increase the number of detected HCV-positive individuals. A systematic review and meta-analysis have shown that expansion of HCV testing services with the use of PoCHCV RNA and reflex HCV RNA viral load testing to lower-level settings such as primary care and harm reduction programmes in the community, performed by appropriately trained non-specialist physicians and nurses could produce a much shorter turnaround time needed between HCV testing and initiation of treatment, compared to laboratory-based standard-of-care testing [42]. Consequently the WHO has recommended PoCHCV RNA viral load testing as an alternative strategy to laboratory-based viral load testing [43].

Our study also pointed out that linkage-to-care at HCV treatment centres being supported by a written protocol or guidelines has increased in the last observational year to half of the centres reporting on it. Simplified linkage-to-care can increase HCV treatment uptake [44]. It has been shown for the population of PWID, that provider coordination of integrated care together with patient education and peer navigation, as well as the use of PoCand dry blood spot tests can improve not only HCV screening uptake, but also linkage to HCV care [44, 45].

Continuum of HCV care includes also liver fibrosis assessment by non-invasive procedures which has been reported to be available also outside healthcare settings in some cities. However, over the observational period a stagnation or even a decreasing trend in proportion of cities reporting such possibilities was noted for all settings. When managing PWID for hepatitis C, the integration of the disease severity assessment within the so called one-stop-shop intervention has also been shown to increase DAA treatment uptake and reduce time to HCV treatment initiation [44]. The assessment of fibrosis stage in hepatitis C is crucial for further follow-up after achieving HCV cure since a certain proportion of patients with advanced liver disease prior to HCV treatment can progress to cirrhosis (in 13% with a stage of fibrosis F3), and are at increased risk of developing liver cancer [46]. Therefore, for such individuals regular lifelong screening for early detection of liver cancer is crucial and implementation of further interventions is needed. The 2023 C-EHRN monitoring report revealed that in 63% of included cities such monitoring practice was in place for PWID whereas 23% of cities reported its unavailability [47].

The settings for HCV treatment in the cities included in our study have largely remained similar over the examined period, with clinical settings (infectious diseases specialists predominating over gastroenterology specialists) consistently being the most common. However, compared to the year 2020, in 2023 an increase in the number of cities reporting HCV treatment at general practitioners was detected. A notable increase has been detected in accessibility to DAA treatment within prisons during the peak of the Covid-19 pandemic. Comparing the last two observational years, in the year 2023 a slight increase in DAA treatment accessible at harm reduction services/community centres was noted, being of vital importance since specialist clinics are usually not easily accessed by PWID. Studies have shown that HCV treatment in PWID that was integrated within drug treatment clinics and primary care settings was superior to standard treatment performed by specialist clinicians regarding increased uptake of treatment and shorter time to treatment initiation, while cure rates were comparable [48, 49].

Taken together, the data obtained in our study for HCV continuum-of-care in PWID most probably immensely reflect the period of the Covid-19 pandemic that coincided with the majority of the observational period in this study (a period between 2020 and 2022). Several studies have clearly shown the negative influence of the Covid-19 pandemic on HCV prevention and testing services, as well as the provision of community-based services [11]. Besides, studies revealed that all steps in the HCV cascade-of-care performed at the clinical settings have been hampered by the Covid-19 pandemic, with a comparable impact across different centres confirming the major effect of the Covid-19 pandemic on global viral hepatitis elimination programs. Restrictions included limited access to HCV screening, counselling, confirmatory testing, and DAA treatment [41, 50]. To overcome those restrictions, some new and innovative strategies were implemented, such as virtual appointments, and alternative modes of medication delivery, while the campaigns for outreach testing in the community and self-testing approaches were least frequently reported, indicating missed opportunities to address identified reductions in testing.

By comparing the situation of HCV continuum-of-care at the beginning of the Covid-19 pandemic (2020) with the year following the Covid-19 pandemic (2023), in our study a decrease was reported for HCV testing and non-invasive liver assessment performance, whereas a stagnation or an increase in the number of cities reporting HCV treatment outside the specialist settings was noted which is in line with the specificities of decentralisation during the Covid-19 pandemic. Taken together the dynamic and trends over the last two observational years and the final situation after the end of the Covid-19 pandemic, improvement was observed across several dimensions in many cities, rebounding after the years of the Covid-19 pandemic. HCV-testing services seemed to have improved in many cities and showed a positive dynamic in nearly all the settings inside and outside the healthcare system. While non-invasive diagnostic procedures for assessing the stage of liver disease presented a slightly negative trend in being performed outside the healthcare system, hepatitis C treatment services performed at harm reduction services/community centers slightly increased. These observations may point out that the influence of the Covid-19 pandemic restrictions on HCV testing performance within healthcare settings has most probably been slowly vanishing [41, 51], while rearrangement of HCV care with decentralization of treatment facilities outside healthcare system due to the Covid-19 pandemic remained also in a post-pandemic era.

Harm reduction organisations play an important role in addressing HCV infection in PWID by implementation of evidence-based harm reduction programmes and raising awareness concerning HCV interventions including HCV testing as well as treatment at the service providers' own site [52]. High coverage of needle/syringe programmes together with OAT has been associated with a 74% decrease in the risk of HCV transmission [53]. During the four-year observational period harm reduction organisations from many cities reported on several limitations and barriers including lack of funding, political support, staff, recognition, as well as the lack of integration of harm reduction services with the healthcare system. However, there was a notable deviation in 2022 with limitations reported from less cities compared to other years. In line with the Covid-19 pandemic and barriers in providing continuum-of-care services, the transient empowerment of harm reduction organisations and the need for their increased operation in the last phase of pandemic might be a realistic and well accepted explanation. Future efforts should be oriented towards increasing access to harm reduction organizations, since so far, even in countries with high OAT coverage and needle/syringes programmes less than one percent of PWID have access to them, particularly due to political resistance, criminalization of drug use, discrimination and stigma [54].

Last, but not least, over the study period the importance of stigma and discrimination in managing PWID with HCV infection has echoed from many countries and cities throughout the treatment policies as well as the cascade of care. In the last observational year, all but five respondents revealed, that stigma and discrimination towards PWID was present in their cities at different points of HCV care, most commonly occurring at gastroenterology clinics, general practitioners and prison settings, whereas only three respondents reported them happened also at harm reduction services/community centres. Results of studies suggest that stigma related to drug use may play a role in HCV transmission and impede efforts to achieve HCV elimination, thus greater efforts are needed to decrease stigma associated to drug use [55].

The most important limitation of this study is the inclusion of one stakeholder recruited from the C-EHRN database of FP harm reduction CSO who was not necessarily profoundly familiar with the respective governmental policy for HCV management, yet was perfectly familiar with HCV interventions and barriers at the local level. Even though the reported information might not be representative for a country per se, it may reflect the diversity of situations within one country, most commonly being dependent upon the city level approach. Of note, the validation of given responses by cross-reference with the current official policies was not provided. However, to overcome this obstacle consultations with external experts were performed, and the responses were placed on the city level. Since the city level perspective is local and the situation might be heterogeneous across a given country, in the future the perspectives and experiences of various civil society stakeholders should be explored and combined.

The major advantage of this study is the real-life perspective given over a four-year progress of HCV continuum-of-care in PWID. In particular cities, it was the CSO that reported on a persistent gap noticed between the official policy on HCV interventions for PWID and their implementation in a real-life situation revealing that this well-known challenge needs to be addressed on the local as well as on the national level, and solutions need to be found as soon as possible.

In the future, the continuum of HCV care for PWID will have to include also a sustainable re-testing for those who remain at risk for HCV infection after cure or spontaneous viral clearance; re-treatment for those who re-tested HCV RNA positive needs to represent a standard-of-care. While in the interferon-based treatment era HCV reinfections were rarely detected, in the era of DAAs they became one of the major barriers on the way to complete HCV elimination, particularly in PWID and incarcerated people in whom the reported rates of up to 20 per 100 person-years and even higher were reported [56–59].

Besides, the introduction of DAAs has led to a HCV cure of virtually all treated patients, exposing the need for performing post-treatment specialized follow-up for those at further risk for developing primary liver cancer even after successful HCV treatment [60]. Only in patients without advanced liver disease and/or certain comorbidities HCV infection can be considered as definitively cured, while in those with advanced fibrosis or cirrhosis liver cancer development has been reported at the rate of 0.5–2.1 per 100 person-years [61]. Early detection of liver cancer is beneficial since it increases the possibility of a curative treatment and may prolong the survival outcome (62).

Taken together both reasons for not dismissing every HCV cured PWID calls for expanding the continuum of HCV care for certain groups of PWID, and urges the future monitoring of progress towards HCV elimination in PWID to include questions on the availability of HCV re-testing and HCV re-treatment, as well as questions on the availability of life-long clinical follow-up after achieving HCV cure.

## Conclusions

This is the first survey presenting trends in the policy and real-life situation of HCV management in a cascade-of-care for PWID over a four-year period, presented by CSOs. It is most probably of significant importance that the results were heavily influenced by the Covid-19 pandemic which coincided with the observational period, however the overall interpretation of a four-year development presents re-establishment after deprivations and restrictions in services and other activities related to the pandemic. Despite clear improvements in policies and practices to eliminate HCV among PWID, achieved in many cities and countries, the overall progress remains insufficient across Europe. Therefore, further improvements in already existing interventions of a continuum-of-care for PWID are needed including the activities for decentralisation, task sharing and particularly patient oriented care, empowered by de-stigmatisation. Besides, an expansion of a continuum of HCV care by adding HCV re-testing, HCV re-treatment and clinical follow-up after HCV cure for certain groups of PWID needs to be immediately introduced.

The data monitoring progress in developments toward HCV elimination, provided by real-life observers from C-EHRN FPs, may serve as an important source of missing information to the WHO GHSS, particularly by providing valuable evidence in assessing the sufficient implementation of effective interventions to eliminate HCV among PWID. The involvement of all stakeholders in monitoring progress in HCV response may provide the most complex map of achievements and deficits that need to be solved on the way to accomplish the WHO GHSS.

The C-EHRN-led monitoring of HCV management in PWID, performed by the CSOs will continue by providing annual reports, using reformulated questionnaires adapted to the current situation, standard-of-care, and needs of data on the local, as well as national level.

## Data Availability

No datasets were generated or analysed during the current study.

## Abbreviations

HCV: Hepatitis C virus
PWID: People who inject drugs
WHO: World Health Organisation
C-EHRN: Correlation-European Harm Reduction Network
CSO: Civil society organisations
DAAs: Direct-acting antivirals
OAT: Opioid agonist treatment
PoC: Point-of-care
EASL: European Association for the Study of the Liver
